# Supplementary material for: Mechanisms governing the pioneering and redistribution capabilities of the non-classical pioneer PU.1
Source: Nat Commun. 2020 Jan 21;11:402. doi: 10.1038/s41467-019-13960-2 (PMC6972792; doi:10.1038/s41467-019-13960-2)
Supplement: Supplementary file 7 — Source data [file 41467_2019_13960_MOESM7_ESM.zip › Source_Data/FigureS2/FigureS2B_MotifScanOutput/KmeansCluster7/homerResults/motif55.similar.html]

motif55

## Information for motif55

A
C
T
G
A
T
C
G
A
T
C
G
A
T
G
C
A
G
T
C
A
C
G
T
C
A
T
G
A
T
C
G
A
C
G
T
A
C
T
G
C
T
A
G
A
C
T
G
  
Reverse Opposite:  

A
G
T
C
A
G
T
C
A
G
T
C
C
G
T
A
A
T
G
C
G
T
A
C
C
G
T
A
A
C
T
G
A
T
C
G
A
T
G
C
A
T
G
C
G
T
A
C
  

|  |  |
| --- | --- |
| p-value: | 1e-20 |
| log p-value: | -4.749e+01 |
| Information Content per bp: | 1.922 |
| Number of Target Sequences with motif | 33.0 |
| Percentage of Target Sequences with motif | 1.03% |
| Number of Background Sequences with motif | 33.1 |
| Percentage of Background Sequences with motif | 0.08% |
| Average Position of motif in Targets | 97.8 +/- 70.2bp |
| Average Position of motif in Background | 116.4 +/- 47.2bp |
| Strand Bias (log2 ratio + to - strand density) | 0.4 |
| Multiplicity (# of sites on avg that occur together) | 1.00 |
| Motif File: | file (matrix) reverse opposite |

### Similar de novo motifs found

|  |  |  |  |  |  |  |  |
| --- | --- | --- | --- | --- | --- | --- | --- |
| Rank | Match Score | Redundant Motif | P-value | log P-value | % of Targets | % of Background | Motif file |
| 1 | 0.819 | C T A G A C T G A T C G C T A G A G T C G T A C A C G T A C T G A C T G A C G T | 1e-14 | -32.779859 | 1.46% | 0.33% | motif file (matrix) |
| 2 | 0.869 | C T G A A C T G C T A G A T C G A C T G A G T C A G T C A C G T A C T G A T C G A C G T A C T G A C T G | 1e-13 | -30.370710 | 0.40% | 0.01% | motif file (matrix) |
